# Bioresorbable Polyester Coatings with Antifouling and Antimicrobial Properties for Prevention of Biofilm Formation in Early Stage Infections on Ti6Al4V Hard-Tissue Implants

**DOI:** 10.1021/acsabm.4c00832

**Published:** 2024-07-22

**Authors:** David Zermeño-Pérez, Hamza Chouirfa, Brian J. Rodriguez, Thomas Dürig, Patrick Duffy, Tadhg Ó Cróinín

**Affiliations:** † Ashland Specialties Ireland Ltd., Mullingar N91 F6PD, Ireland; ‡ School of Physics, University College Dublin, Dublin Dublin 4, Ireland; § Ashland Wilmington Centre, Wilmington 19808, Delaware, United States; ∥ School of Biomolecular and Biomedical Science, University College Dublin, Dublin Dublin 4, Ireland

**Keywords:** poly(d,l-lactide), methyl ether poly(ethylene glycol), medical coatings, biofilm, antimicrobial, antifouling, silver sulfadiazine

## Abstract

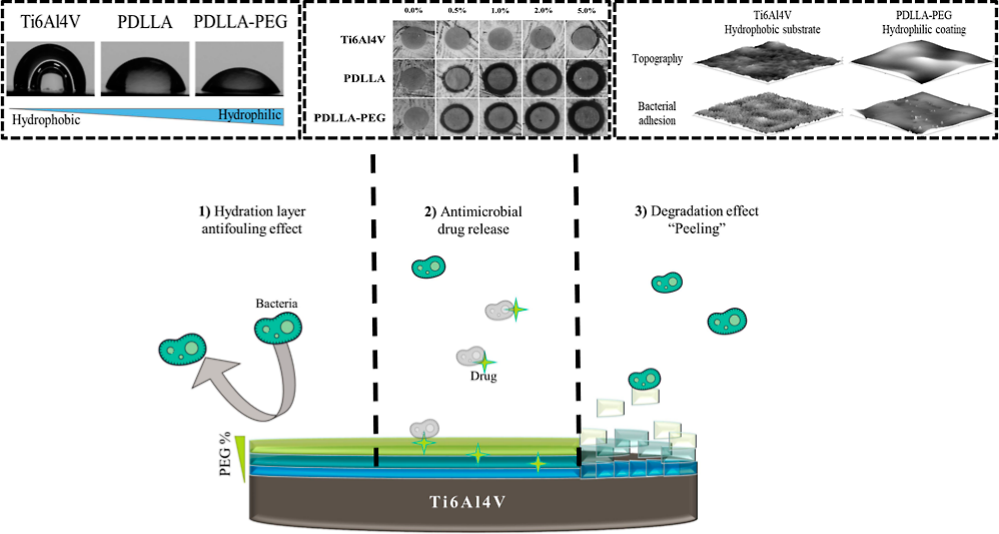

Implants made from titanium are used as prostheses because of their biocompatibility and their mechanical properties close to those of human bone. However, the risk of bacterial infection is always a major concern during surgery, and the development of biofilm can make these infections difficult to treat. A promising strategy to mitigate against bacterial infections is the use of antifouling and antimicrobial coatings, where bioresorbable polymers can play an important role due to their controlled degradability and sustained drug release, as well as excellent biocompatibility. In the present study, poly(d,l-lactide) (PDLLA) and poly[d,l-lactide-*co*-methyl ether poly(ethylene glycol)] (PDLLA–PEG) were studied, varying the PEG content (20–40% w/w) to analyze the effectiveness of PEG as an antifouling molecule. In addition, silver sulfadiazine (AgSD) was used as an additional antimicrobial agent with a concentration ≤5% w/w and incorporated into the PEGylated polymers to create a polymer with both antifouling and antimicrobial properties. Polymers synthesized were applied using spin coating to obtain homogeneous coatings to protect samples made from titanium/aluminum/vanadium (Ti6Al4V). The polymer coatings had a smoothing effect in comparison to that of the uncoated material, decreasing the contact area available for bacterial colonization. It was also noted that PEG addition into the polymeric chain developed amphiphilic materials with a decrease in contact angle from the most hydrophobic (Ti6Al4V) to the most hydrophilic PDLLA–PEG (60/40), highlighting the increase in water uptake contributing to the hydration layer formation, which confers the antifouling effect on the coating. This study demonstrated that the addition of PEG above 20% w/w and AgSD above 1% w/v into the formulation was able to decrease bacterial adherence against clinically relevant biofilm former strains *Staphylococcus aureus* and *Pseudomonas aeruginosa*.

## 1 Introduction

Many pathogenic agents, including bacteria, fungi, and viruses, can infect the human body. The most prevalent kind of acute and chronic illnesses causing morbidity worldwide are bacterial infections. Antibiotic-resistance in clinically relevant pathogens is increasing leading to an alarming rise in the prevalence of incurable bacterial infections.^1^ There are two distinct states of bacteria: “planktonic,” which are individual bacterial cells, and “biofilm,” which are bacterial agglomerates attached to a surface. The bacterial biofilms are known for their synergistic communal behavior and the use of a protective barrier composed mainly of exopolysaccharide, although other elements can be present, such as lipopolysaccharides, proteins, lipids, glycolipids, and nucleic acids. This barrier protects the bacteria against the organism’s immune system and from external agents such as antibiotics.^2^ Biofilm-forming bacteria are known to cause chronic infections characterized by persistent inflammation and tissue damage. Most importantly, these biofilms have been found on medical devices and hospital surfaces.^3^ Most clinically relevant bacterial species linked to healthcare-associated infections (HAIs) were reviewed by Kamaruzzaman et al., along with the potential sites of infection where biofilms cause disease, as well as diagnostics and treatments that are available.^4^ Medical devices can harbor biofilms of both Gram-positive and Gram-negative bacteria; however, the most prevalent types of these bacteria include *Staphylococcus aureus*, *Staphylococcus epidermidis*, *Enterococcus faecalis*, *Streptococcus viridians*, *Escherichia coli*, *Klebsiella pneumoniae*, *Proteus mirabilis*, and *Pseudomonas aeruginosa*. Of these, it is estimated that Gram-positive *S. aureus* and *S. epidermidis* account for 40–50% of infections in prosthetic heart valves, 50–70% of catheter biofilm infections, and 87% of bloodstream infections;^5^ meanwhile, the opportunistic Gram-negative *P. aeruginosa* contributes to >90% of biofilm infections on endotracheal tubes giving rise to ventilator-associated pneumonia.^6^ According to data from 2018 from the European Centre for Disease Prevention and Control (ECDC), approximately 3.8 million people in EU member states, Norway, and Iceland contracted HAIs, with an estimated 90,000 deaths reported annually.^7^

Francolini et al. highlight that according to the staged process of biofilm formation, possible antibiofilm strategies should be based on inhibition of microbial adhesion to the surface and colonization, interference with the signal molecules modulating biofilm development, and disaggregation of the biofilm matrix.^8^ Silver and copper-based molecules have long been used as antimicrobial agents, used as nanoparticles (NPs) or by ion release, for the protection of medical devices and to keep low bioburden levels on hospital surfaces.^9^ Copper is the most utilized element due to its low cost, availability, and wide range of applications, but it is important to consider two factors: toxicity of these elements as NPs and ion internalization on tissue outside of the site of treatment, which contributes to the development of secondary effects and, in the presence of an infection, bacterial resistance.^10^ The limitation with copper deposition by various coating methods is that excessive copper is highly dangerous, and the long-term performance of antimicrobial coatings is not addressed in laboratory studies, as well as whether these metals can cause premature host cell death.^11−13^

Silver sulfadiazine (AgSD) has largely been marketed in a formulation of 1% AgSD cream as Flamazine (Smith and Nephew, Hull) and as a cream with 1% AgSD in combination with 0.2% chlorhexidine digluconate as Silvadene (Monarch Pharmaceuticals, Tennessee, USA) in the North American region.^14^ It has shown a mechanism of inhibition similar to that seen with silver NPs, proving to be extremely effective as a wound healing and antimicrobial agent. AgSD is classified within the sulfa antibiotics’ family by the substitution of a hydrogen atom by a silver atom in the sulfadiazine molecule; the combinatory antimicrobial effect of silver and sulfa groups is greatly enhanced in coating applications.^15^

Given the hydrophobic nature of microbial surfaces, bacterial adherence can be prevented by covering the device surface with a hydrophilic coating. In the past 20 years, research efforts have been concentrated on the surface adsorption or material impregnation of one or several antimicrobial compounds to prevent the proliferation of previously attached and/or colonized bacteria.^16^ It has been shown that hyaluronic acid^17^ and poly-*N*-vinylpyrrolidone,^18^ which were initially employed to coat silicon shunts and polyurethane catheters, respectively, are examples of hydrophilic polymers that effectively decrease Gram-positive *S. epidermidis* adherence. Numerous hydrogel coatings, particularly for ureteral stents, have also been produced due to their enhanced degradability and controlled release of hydrophilic antibiotics because of their high water uptake potential, as well as their capacity to decrease bacterial adherence due to their hydrophilic qualities.^19,20^

Aliphatic polyesters such as poly(lactide), poly(glycolide), poly(caprolactone), and copolymers thereof have been widely researched and used in biomedical applications due to their inherent biodegradability and biocompatibility, which has granted these materials long-standing approval by the Food and Drug Administration (FDA) and the European Medicines Agency (EMA).^21−23^ In addition, synthesis can be easily controlled, and polymer characteristics can be tailored to provide a family of materials with a relatively wide range of physical properties and degradation rates. Moreover, these polymers can be easily functionalized or loaded with antimicrobial agents for use as coatings for hard-tissue implants. Al-doped ZnO (AZO) coatings on biopolymer poly(l-lactide) (PLLA) were generated as a green antibacterial nanocomposite, with demonstrated strong antibacterial activity against two Gram-negative bacteria (*E. coli* and *Pseudomonas sp.*) and two Gram-positive species (*S. aureus* and *Bacillus sp.*).^24^ Poly(lactide-*co*-glycolide) (PLGA) and poly(d,l-lactide) (PDLLA) have been studied as antimicrobial coatings, where polymer blends with β-TCP and vancomycin show inhibition of methicillin-resistant *S. aureus* (MRSA)^25^ and PDLLA loaded with gentamicin sustained drug release for 1 week and achieved full degradation in six months while protecting implants from infections without having adverse effects.^26^ However, these polyesters are relatively hydrophobic with degradation times ranging from 6 to 24 months and often require incorporated antimicrobial agents to be properly used as antimicrobial coatings; hence, copolymerization with hydrophilic molecules is being studied to enhance degradability and create amphiphilic molecules suitable as short-term antifouling and antimicrobial coatings. Poly(ethylene glycol) (PEG) has been widely used in combination with bioresorbable polymers as a stealth molecule to prevent phagocytosis. PEG has an inherent hydrophilic nature, which creates a hydrophilic barrier with antibiofouling properties, preventing interactions with blood components, bacterial cells, substrates, and biomaterials.^27,28^

**Figure 1:**
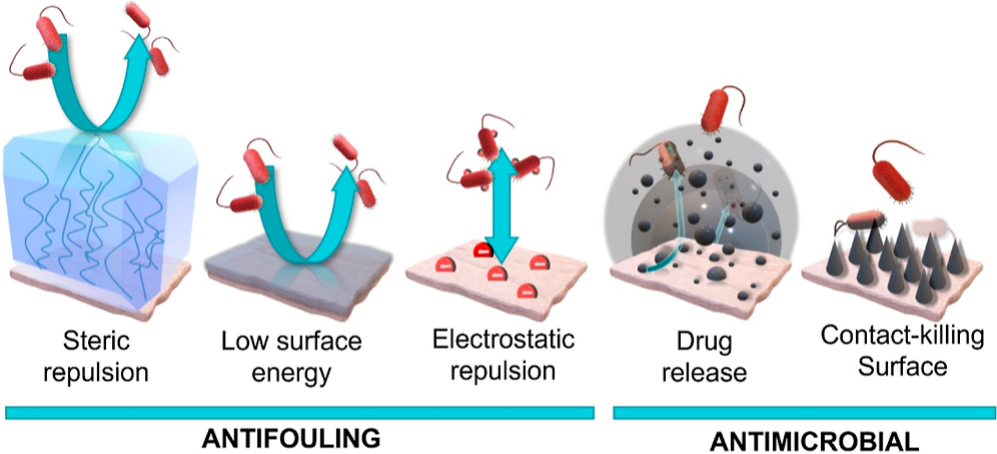
Diagram of antifouling and antimicrobial coating strategies applied to polymers.

The aim of this study was to formulate PEGylated bioresorbable polyesters with different degrees of hydrophilicity. Polymers were assessed as unloaded as a drug-free antifouling coating and drug-loaded with AgSD as an antibiofilm coating (Figure 1). All samples were tested against clinically relevant species of Gram positive (*S. aureus*) and Gram negative (*P. aeruginosa*) pathogens known to be associated with biofilm-mediated infections on healthcare devices.

## 2 Materials and Methods

### 2.1 Materials

d,l-Lactide monomer was acquired from Corbion (PURASORB DL, Netherlands); methyl ether PEG *M*_n_: 5000 Da (mPEG_5000_), chloroform ≥99.8% (CHCl_3_), tetrahydrofuran (THF) stabilized with 250 ppm of BHT ≥99.8% (THF), tin(II) 2-ethylhexanoate, dodecanol ≥99%, silver(I) sulfadiazine (AgSD) > 98%, lysogeny broth (LB) and agar, and phosphate buffered saline (PBS) were purchased from Sigma-Aldrich (United Kingdom); titanium/aluminum/vanadium (Ti6Al4V)—grade 5 (ASTM B265) discs with 10 mm diameter and 1 mm thickness were purchased from Goodfellow (United Kingdom).

### 2.2 Bacterial Strains

*S. aureus* (SH1000) and *P. aeruginosa* (PA01 + GFP)^29^ were used as test microorganisms due to their clinical relevance as infectious biofilm formers on medical devices. All strains were obtained from the biobank of Dr. Tadhg Ó Cróinín at the School of Biomolecular and Biomedical Science, University College Dublin. Bacteria were prepared and cultured in fresh LB agar plates from frozen stocks. For liquid media preparation, one loopful of bacteria was inoculated in a test tube with LB media and grown in an orbital incubator under shaking conditions at 200 rpm and 37 °C for 24 h. The optical density of these solutions was measured by using a UV-spectrometer. These cultures were diluted in fresh LB broth to an OD_600_ of 0.1 (∼5 × 10^7^ cells/mL) as starting inoculation concentration for antifouling and antimicrobial tests.

### 2.3 Synthesis of Bioresorbable Polymers

d,l-Lactide monomer, mPEG_5000_ or dodecanol as initiator, and tin(II) 2-ethylhexanoate were dried inside a stainless-steel reactor vessel at 45–60 °C in an inert environment for 1 h. Ring opening polymerization of PDLLA (12 kDa), PDLLA–PEG 80:20 (16 kDa), and PDLLA–PEG 60:40 (7.5 kDa) was carried out in bulk by increasing the temperature of the vessel to 110–150 °C and maintaining constant stirring.^30^ Each polymerization was carried out for 5–8 h in an inert environment, and samples were taken hourly to determine reaction kinetics. The target *M*_n_ of each reaction was calculated using the following equation
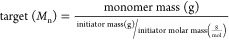
2.3.1

The PEG content (wt %) was calculated by the following equations
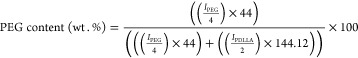
2.3.2In which *I*_PEG_ is the integration peak at 3.71–3.56 ppm, while *I*_PDLLA_ is the integration peak at 5.28–5.09 ppm.

#### 2.3.1 Silver Sulfadiazine Drug Loading

To blend the polymer with the antimicrobial agent, 2 g of polymer was dissolved for an hour in 10 mL of chloroform. AgSD was added to the polymer solution in 0, 1, 2.5, and 5% w/w, creating several mixtures with increased drug load.

### 2.4 Material Characterization

#### 2.4.1 Proton Nuclear Magnetic Resonance

Proton nuclear magnetic resonance (^1^H NMR) was utilized to determine the monomer conversion, PEG content, and chemical composition of the synthesized polymers. A 400 MHz Bruker NMR machine was used to obtain the spectra, which were analyzed and processed using the MestReNova software and reported in parts per million (ppm) relative to the response of deuterated chloroform (CDCl_3_) used as a solvent (7.26 ppm). Solutions were prepared dissolving 20 mg of polymer in 700 μL of CDCl_3_, and each solution was then filtered into NMR sample tubes.

#### 2.4.2 Gel Permeation Chromatography

Samples were prepared by dissolving the polymers in THF at a concentration of 8–10 mg/mL, followed by filtration. Gel permeation chromatography (GPC) measurements were performed using an Agilent triple detector system with an Agilent Technologies column (two 8 × 300 mm 1000 Å and two 8 × 300 mm 10,000 Å plus guard), where filtered THF was used as the mobile phase with a flow rate of 1.0 mL/min. Weight-average molecular weight (*M*_w_), number-average molecular weight (*M*_n_), and polydispersity index (D̵) data were analyzed by using the refractive index peak (conventional method) using polystyrene standards as calibration curve, which generated relative values.

#### 2.4.3 Water Content

Water content characterization was carried out through Karl Fischer titration using Titrando 874/851 (Metrohm, United Kingdom) to analyze the water content of the polymers after synthesis. 300 mg of polymer was weighted and placed in glass vials and crimped with aluminum-septum cap. The polymer samples were melted at 150 °C. The end point was detected electrochemically until drift reached a plateau.

#### 2.4.4 Degradation Study

Polymer discs (10 mm diameter and 1 mm thickness) were prepared by melt casting using a silicon mold with 500 mg of polymer in each well. Melting of the polymer granules was performed within a vacuum oven at 110 °C for 30 min under −5 mPa. Samples were incubated in 10 mL of PBS (pH = 7.2 ± 0.2) in a heated incubator (37 ± 1 °C) at a shaking rate of 100 rpm. The study was performed for 49 days, where at a specific interval, the samples were washed with distilled water and dried in a vacuum desiccator until a constant weight is obtained. The sample weight and pH of the degradation media were recorded before and after incubation. Medium was renewed with fresh PBS depending on the water uptake of the samples to keep the volume constant. Sample degradation was evaluated by the weight loss of the samples; meanwhile, water uptake was calculated by the wet and dry weight calculated after each interval according to the following equations
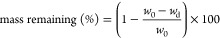
2.4.4.1
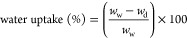
2.4.4.2where *w*_0_ is the initial weight of the samples before incubation, *w*_w_ is the wet weight of each sample after incubation, and *w*_d_ is the dry weight at each incubation time.

### 2.5 Polymer Coating Application

Ti6Al4V discs were cleaned through sonication with acetone and 2-propanol and left to dry before coating application. Polymer solutions were made fresh at 20% w/v in chloroform before each batch of coated samples. Spin coating was performed using static dispensing technique due to the viscosity of the solutions; 75 μL was used to cover homogeneously the area of the discs (78.50 ± 0.05 mm^2^) at a rotational speed of 6000 rpm for 30 s per layer. Samples were left to dry overnight before testing.

Multilayer coating is a gradient coating of the polymers used in this work, where PDLLA was coated as the initial layer in contact with Ti6Al4V, followed by PP8020 and PP6040. The trilayer coating comprises the multilayer sample seen across this article.

### 2.6 Surface Characterization

#### 2.6.1 Contact Angle

The wettability behavior (degree of hydrophilicity) of the polymeric coatings on Ti6Al4V discs was evaluated by measurement of the static contact angle with a goniometer (Ossila, The Netherlands) using an uncoated Ti6Al4V as a control. Video recordings of the water droplets (10 μL) in contact with the coatings were captured at 0.5 frame/s during the lapse of one min; the results were processed by edge detection using the Ossila Contact Angle 4.1 software.

#### 2.6.2 SEM–EDS

Scanning electron microscopy (SEM) was conducted using a Thermofisher Axia ChemiSEM operating at an accelerating voltage between 0.5 and 5 kV. Titanium discs spin-coated with polymers were previously dried for 24 h in vacuum desiccator and mounted on carbon tape. Polymers were visualized without a conductive coating.

#### 2.6.3 Topographical AFM

Surfaces were imaged using amplitude modulation atomic force microscopy (AFM) (MFP-3D, Asylum Research) operated with Si probes (NCH, Nanosensors) having a nominal spring constant of 42 N/m and a resonance frequency of 330 kHz. Representative images at 90 μm × 90 μm, 50 μm × 50 μm, and 10 μm × 10 μm were collected in multiple locations to analyze topography and root-mean-square (RMS) roughness. 3D image was flattened to remove sample tilt, and horizontal correction was applied if required.

### 2.7 Antimicrobial Effect

#### 2.7.1 AgSD Drug Diffusion

A release assay was conducted to investigate the diffusion of the AgSD coatings over an extended period in PBS buffer (pH = 7.2 ± 0.2). An appropriate wavelength (290 nm)^31^ was used to identify AgSD; release studies were conducted on polymer coatings containing AgSD, which were prepared in triplicate (*n* = 3) as previously described at the concentrations of 0.0, 0.5, 1.0, 2.0, and 5.0% w/v. The release assay was conducted over 128 h, and samples were taken at time points 0, 2, 4, 8, 24, 48, 72, 96, and 128 h.

#### 2.7.2 Halo Assay

For testing of the antimicrobial effect, a standard disc diffusion test was performed following the protocol outlined in the Kirby–Bauer test.^32^ Spin-coated samples blended with AgSD (at 0.0, 0.5, 1.0, 2.0, and 5.0% w/v) were tested against *S. aureus* (SH1000) and *P. aeruginosa* (PA01 + GFP) using filter paper with AgSD 1.0% w/v in PBS as a positive control.^31^ Plates were incubated for 24 h, and zones of inhibition were measured. Zone diameters were calculated by using ImageJ.

#### 2.7.3 Biological AFM

AFM was also used to evaluate the degree of adhesion and coverage of *S. aureus* (SH1000) and *P. aeruginosa* (PA01) on Ti6Al4V discs and coated samples after 2 h of inoculation. Samples were sterilized with 70% ethanol before inoculation. Bacterial strains were grown in LB media for 24 h at 37 °C under constant shaking of 200 rpm. After growth, liquid cultures were centrifugated at 1000 rpm, and deionized water (dH_2_O) was used to resuspend the bacterial pellet at an OD_600_ of 0.1. The pellet was washed three times in dH_2_O in order to avoid formation of media crystals and prevent bacterial osmotic disruption. 250 μL of bacterial solution in dH_2_O was deposited on top of each sample and incubated for 2 h at 37 °C. After incubation, three washes with dH_2_O were performed to discard planktonic and unadhered bacterial cells. 4% formaldehyde was used to fixate bacterial cells adhered to the coatings before AFM analysis.

#### 2.7.4 Epifluorescent Microscopy

Epifluorescent microscopy was used to analyze the surface of coatings after performing the halo assay with *P. aeruginosa* carrying the green fluorescent protein (PA01 + GFP) to corroborate the viability of the strain after being in contact with AgSD at different concentrations.

### 2.8 Statistical Analysis

The number of samples evaluated is three (*n* = 3) by default unless stated otherwise; for microbiological testing, three biologicals with two technical were applied (*n* = 6). The significant differences (*P* > 0.01) of the results were analyzed via one-way analysis of variance.

## 3 Results

### 3.1 Synthesis of Bioresorbable Polymers

**Figure 2:**
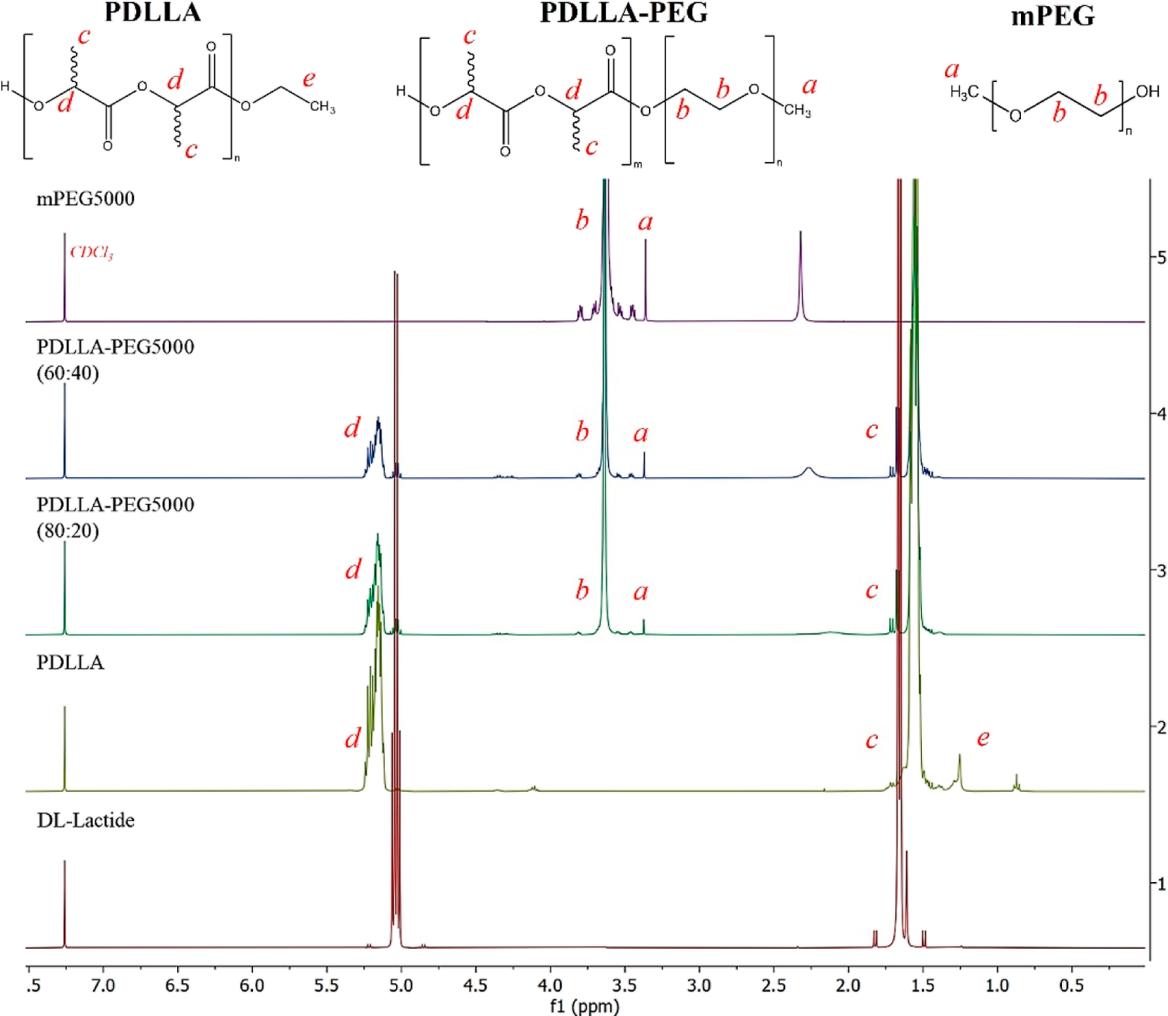
^1^H NMR spectra in CDCl_3_ (7.26 ppm); comparison of d,l-lactide, PDLLA, PDLLA–PEG (80:20 and 60:40), and mPEG_5000_.

The synthesis of PDLLA and mPEG–PDLLA polymers was confirmed by ^1^H NMR. Figure 2 shows the spectral overlay for all of the synthesized polymers and monomer repeat units. PDLLA spectra showed peaks at 1.64–1.56 ppm and 5.28–5.09 ppm, which corresponded to methyl “c” (CH_3_) and methine “d” (CH) protons, respectively, confirming successful synthesis of PDLLA.^33^ PEG was identified at a chemical shift of 3.71–3.56 ppm for methylene (CH_2_) and 3.38–3.35 ppm for methyl (CH_3_) groups.^33,34^ The conversion of monomer to polymer was calculated based on the integration of the CH_3_ and CH signal peaks for the respective polymers and monomers introduced and is reported in Table 1.

**Table 1:** Summary of Polymer Characterization by ^1^H NMR, GPC-THF, and Water Content

polymer	monomer (M)	initiator (I)	molar ratio M/I	target *M*_n_ (kDa)	conv. (%)^a^	*M*_n_^b^ (kDa)	*M*_w_^b^ (kDa)	D̵^b^	PEG content^a^(wt %)
PDLLA (ester-cap)	d,l-lactide	decanol	80:1	12.0	97.9	15.2	28.0	1.8	
PDLLA–PEG_5000_ (80:20)		mPEG_5000_	110:1	16.0	98.2	13.8	21.0	1.5	22.3
PDLLA–PEG_5000_ (60:40)		mPEG_5000_	52:1	7.5	96.0	7.8	10.5	1.3	42.5

a NMR.

b GPC.

Peaks “a” and “b” confirm the presence of PEG in both PDLLA–PEG copolymers (80:20 and 60:40). PDLLA shows peak “e” as the terminal methyl protons, whereas after PEGylation, this peak disappears, which confirms successful copolymerization.

^1^H NMR analyses in DMSO-*d*_6_ were also performed to identify the terminal hydroxyl group (peak “e”) of mPEG_5000_ (4.6–4.4). The disappearance of this peak and the presence of multiple peaks in the region of 4.4–4.0 and 1.0–0.8 are an indication of the ester termination of the polymeric chain, suggesting a positive copolymerization of PDLLA–PEG at different ratios. GPC was also used to confirm polymer structure and unimodal population after synthesis of PDLLA and PDLLA–PEG copolymers. Within the Supporting Information, the data from the DMSO-*d*_6_ NMR (Supporting Information: Figure S1) and GPC (Supporting Information: Figure S2) can be found.

It was possible to confirm successful synthesis of the following polymers and copolymers with the 80:20 and 60:40 ratios. A summary of the characterization results can be found in Table 1. PDLLA–PEG copolymers with 40% PEG content show a decreased molecular weight (*M*_w_) compared to those with 20% PEG as the amount of initiator (mPEG) is increased accordingly.

### 3.2 Degradation Study

As bioresorbable polymers are degraded by hydrolysis, the initial water content was tested to guarantee a starting point with <0.5% wt.

**Figure 3:**
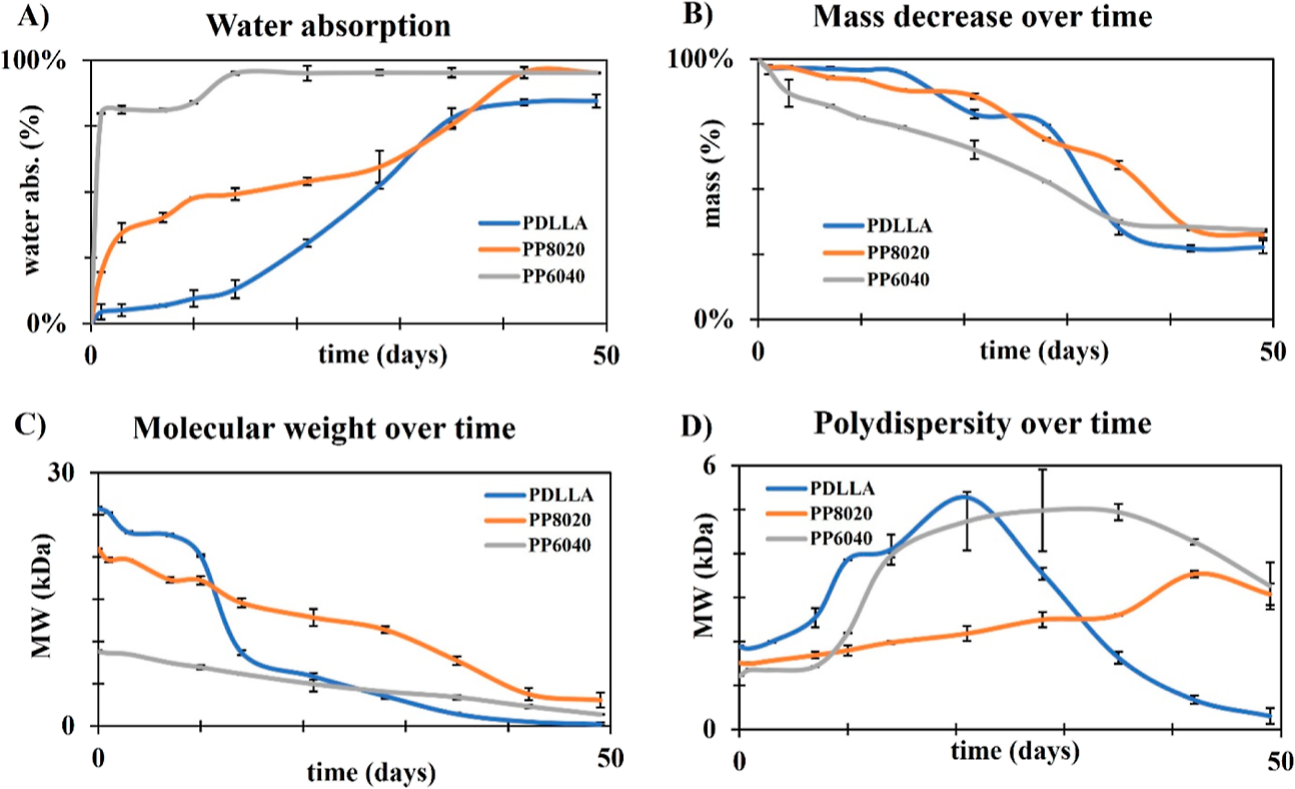
Dynamic hydrolytic degradation study. (A) Water absorption, (B) mass decrease over time (49 days), (C) *M*_w_ decrease, and (D) PDI changes over time (49 days). Data points without standard deviation are shown as an average. Samples were analyzed with *n* = 3 values.

The water absorption and mass decrease are shown in Figure 3A,B, where PDLLA critical mass loss was achieved after 28 days with a steady water absorption and pH decrease (Supporting Information: Figure S3) after the 10th day, reaching a plateau after 35 days. Whereas for the PEGylated versions, there is a clear impact on the early water absorption contributed by the PEG from the first to the seventh day, where PP8020 and PP6040 achieved 40 and 80% water absorption within this time frame. Secondary water absorption point is visible from the 10th day, following a trend of water uptake behavior similar to that of pure PDLLA.

*M*_w_ and PDI changes are shown in Figure 3C,D, where the decrease in *M*_w_ was seen immediately upon immersion in PBS, while significant mass loss did not begin until 2 weeks later, except for PP6040, where the mass loss difference was clear from the third day due to increased PEG content and lower starting *M*_w_. PDLLA *M*_w_ reached a critical point with a decrease of 50% during the second week; meanwhile, PEGylated versions seem to have a steadier decrease of the *M*_w_ overtime in comparison. The loss of structural integrity was an early indication of the critical mass loss, where PP6040 reached the point of total solubilization after 3 days and PP8020 reached solubilization after 14 days. PDLLA did not become completely soluble, but loss of structural integrity was clear after 21 days (Supporting Information: Figure S4).

### 3.3 Contact Angle

**Figure 4:**
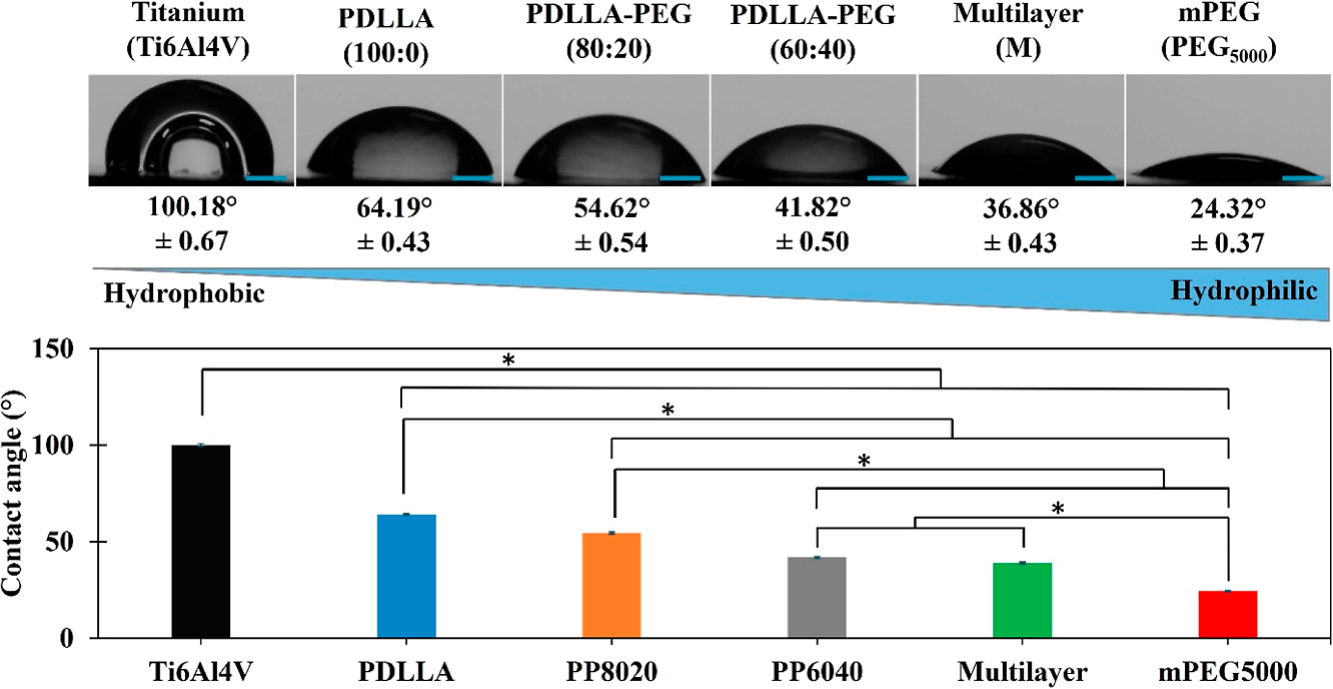
Contact angle analysis on titanium surfaces coated with polymer solutions. Addition of PEG into the polymer chain decreases the contact angle, indicating an increase in surface hydrophilicity. SD shown next to values on images for reference as not clearly seen on graph. 1 mm scale bar used on goniometer images. Samples were analyzed with *n* = 3 and *P* > 0.01 values.

The contact angle allowed us to evaluate the hydrophilicity of the samples by measuring the ability of a liquid to wet the surface of a material. Water contact angle measurements from the polymer coatings were found to decrease with the addition of PEG into the polymer chain, as is shown in Figure 4. Ti6Al4V is known to be hydrophobic^35^ with a contact angle of 100.18°, and PEG to be highly hydrophilic^36^ due to its polarity with a contact angle of 24.32°. Hydrophobic PDLLA (64.19°) when copolymerized using hydrophilic PEG (24.32°) achieves an amphiphilic state, with PDLLA–PEG 80:20 and 60:40 showing a decrease in contact angle of 54.62 and 41.82°, respectively; even though PEG would be considered more hydrophilic, it showed to be fully soluble after 5 min.

### 3.4 Surface Analysis through SEM–EDS

**Figure 5:**
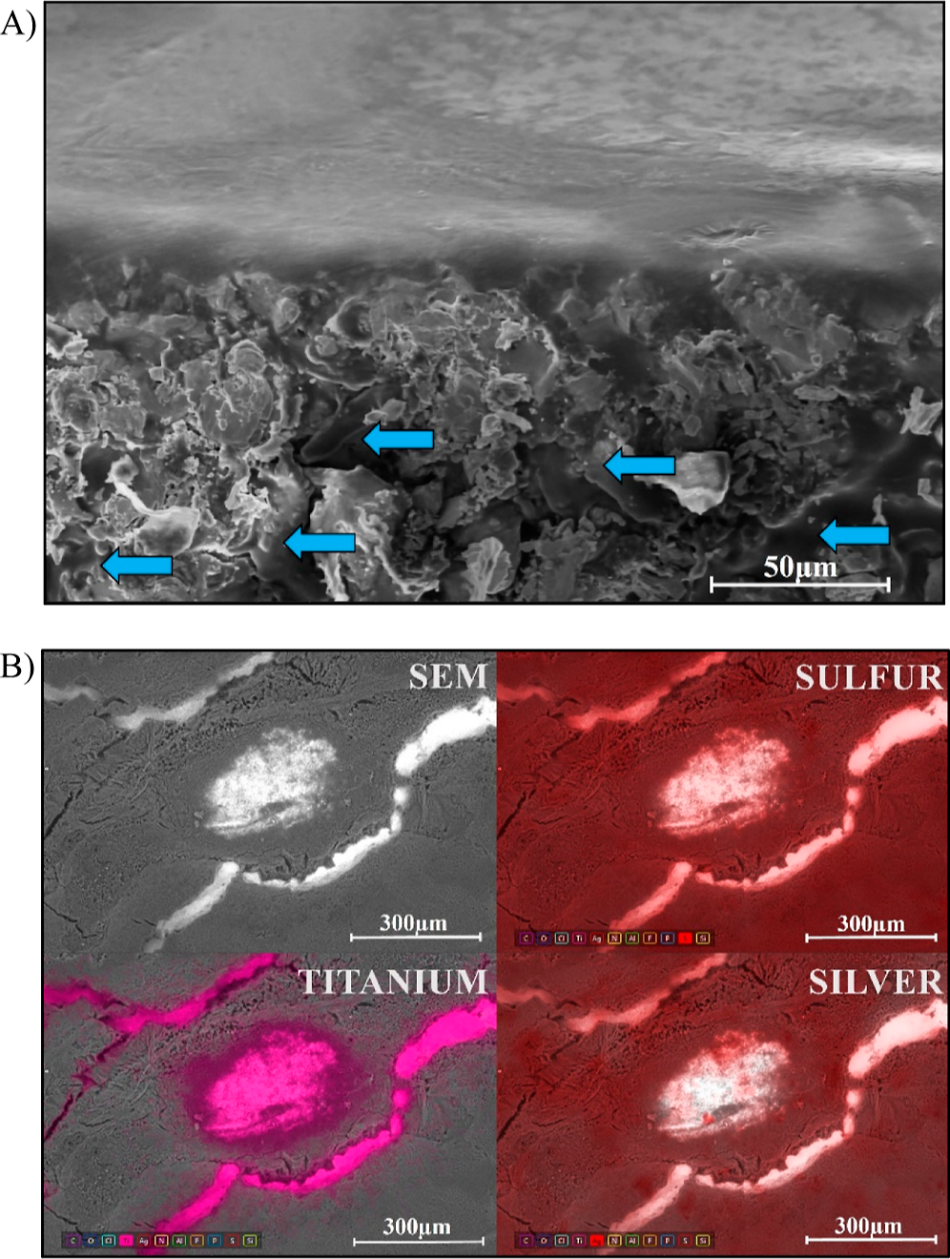
SEM analysis. (A) Tilted SEM (45°) of the polymer filling the topography of Ti6Al4V through the gaps working as anchoring points (arrows), contributing to the smoothening of the surface and adherence to the metallic surface. (B) SEM-EDS of titanium surface with a PDLLA–PEG (80:20) coating with 1% AgSD blended. Purple (titanium) and red (Ag and S elements). Samples were analyzed with *n* = 3 values.

SEM–energy-dispersive spectroscopy (EDS) was performed qualitatively on samples to qualify how homogeneous the coating is and identify physical anchoring points and how intertwined the polymer and the Ti6Al4V were after spin coating (Figure 5A). Half-coated discs are shown in Supporting Information for comparison between surfaces and EDS analysis between titanium (yellow) and the polymeric coating (C in blue and O in red) (Supporting Information: Figure S5). The smoothening effect of the polymeric coating was present on all samples, being able to cover the complicated topography of Ti6Al4V and leave a smooth finish on the surface. Roughness was characterized by an AFM to support this theory. EDS analysis was performed in drug-free and drug-loaded samples with 1% AgSD (Figure 5B). Intentional scars were made on the coating to be able to see the substrate (titanium, purple) as well as identify the presence of the antimicrobial drug AgSD (Ag and S in red), where Ag and S elements are present in the antimicrobial and were found confirming the presence of AgSD homogeneously within the coating.

### 3.5 AFM Antifouling Analysis

**Figure 6:**
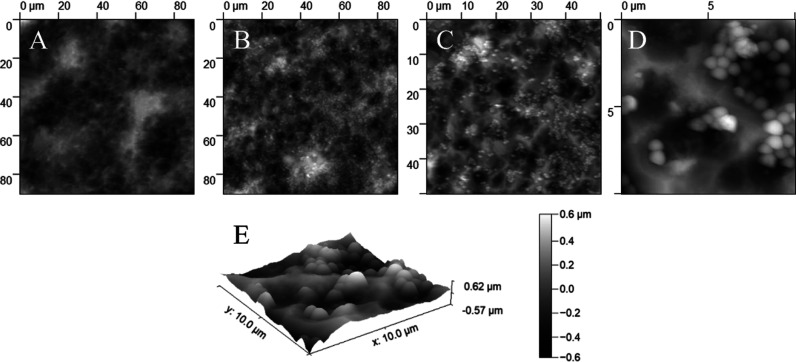
AFM top view of (A) Ti6Al4V disc before incubation for topographical reference and after 2 h incubation with *S. aureus* (SH1000), (B) at 90 μm × 90 μm, (C) 50 μm × 50 μm, and (D) 10 μm × 10 μm with (E) 3D representation of image (D) showing bacterial adherence and confirmation of cell morphology/shape. Z-scale of 1.2 μm was used in all of the images. Samples were analyzed with *n* = 6 values.

To understand the bacterial adhesion and the surface topography preference to the Ti6Al4V grade 5, samples were incubated with *S. aureus* (SH1000) at 0.1 OD_600_ for 2 h and were analyzed with AFM. Images were taken at different locations to see the degree of homogeneity of the bacterial adhesion with bare Ti6Al4 (Figure 6A) used as control. Figure 6B–D shows that the bacterial cells were found to be homogeneous across the surface of the samples. In Figure 6D, it is possible to observe that the individual cells have a predilection to agglomerate and start biofilm formation at the depths and sharp angles of the topography of Ti6Al4V.

**Figure 7:**
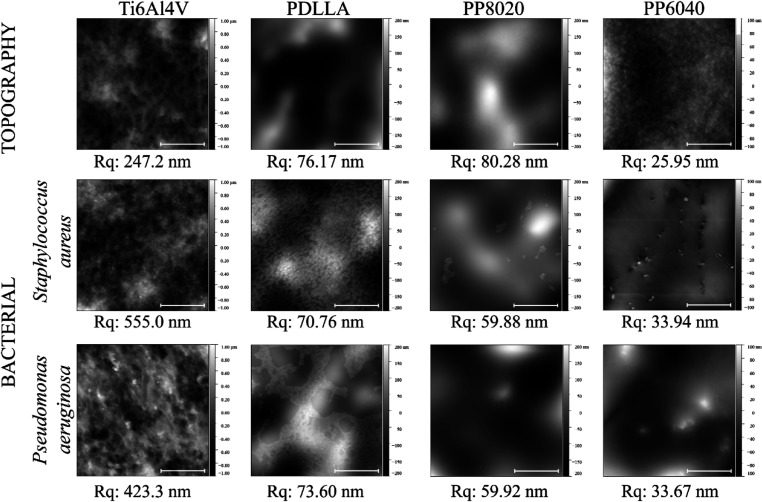
AFM top view of uncoated Ti6Al4V and coated sample discs before incubation for topographical reference and after 2 h incubation with *S. aureus* (SH1000) and *P. aeruginosa* (PA01), first-order flattening was applied to all images. 30 μm scale bar. Samples were analyzed with *n* = 6 values.

When the samples were coated with the amphiphilic polymers (20% w/v), the roughness (*R*_q_) decreased from 247 nm for Ti6Al4V to 76, 80, and 26 nm for PDLLA, PP8020, and PP6040, respectively, as shown in Figure 7. When these coated samples were incubated with *S. aureus* and *P. aeruginosa*, the bare titanium and PDLLA are covered with both bacterial strains, whereas in PP8020 and multilayer, there is a clear reduction in bacterial cells adhered to the polymeric surface.

Images were also processed through second-order polynomial; this was performed to further remove background to increase contrast between substrate and bacterial cells, allowing easier bacterial identification and confirmation (Supporting Information: Figure S6).

### 3.6 Antimicrobial Coating Effect

**Figure 8:**
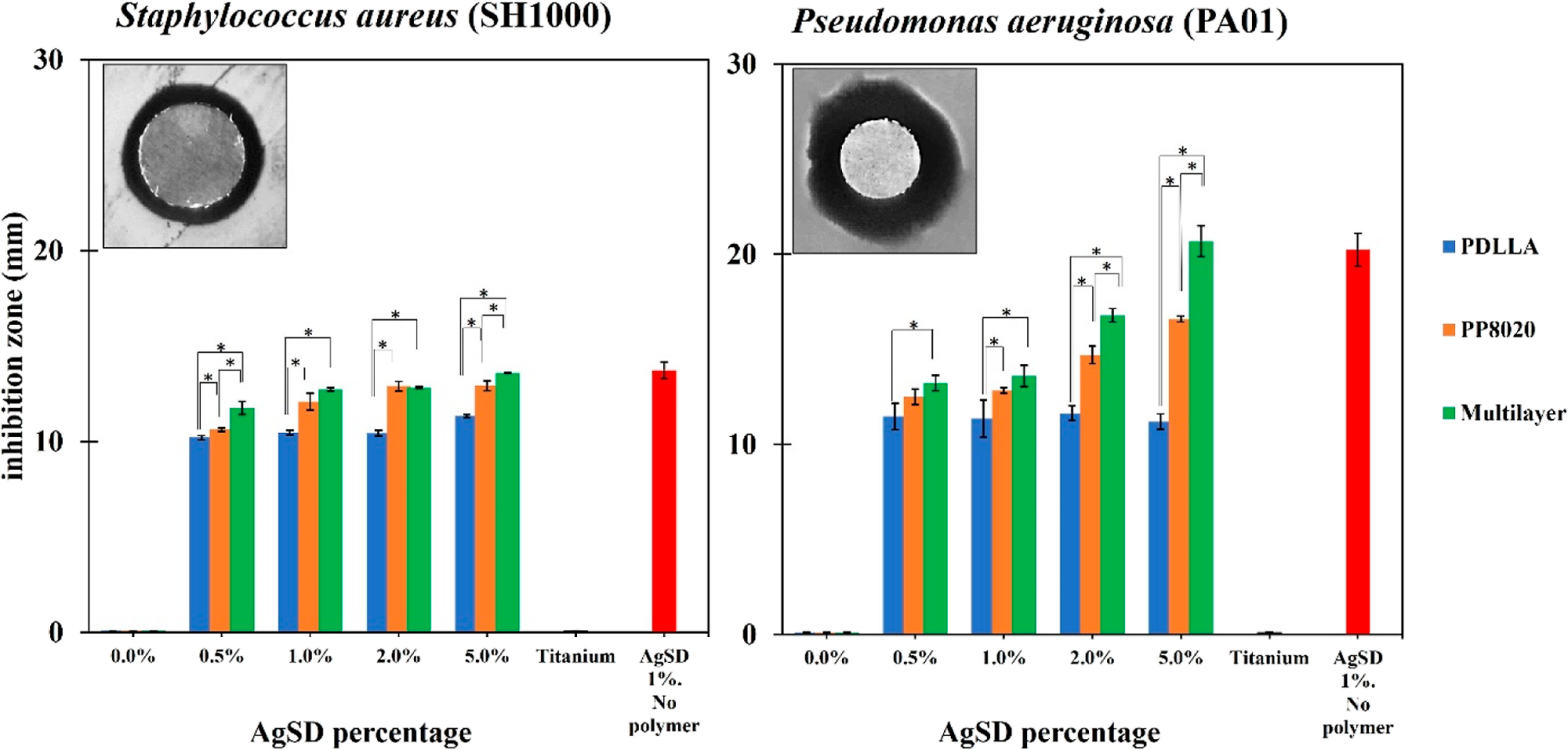
Halo inhibition zones after 24 h on the plated samples. Zone diameters were calculated using ImageJ, using titanium disc (10 mm diameter) as reference. Samples were analyzed with *n* = 6 and *P* > 0.01 values.

Polymer coatings blended with different percentages of AgSD (0.5–5.0% w/v) were tested using a simple Kirby–Bauer test for drug diffusion and inhibition of *S. aureus* and *P. aeruginosa* over 24 h, with the exception of monolayer PP6040 due to early detachment from the substrate. As shown in Figure 8, PDLLA shows no significant difference between any of the concentrations for both strains, except for 5% AgSD with *S. aureus*. Meanwhile, the monolayer PP8020 showed increased antimicrobial effect in comparison with Ti6Al4V. The best antimicrobial effect was shown by the multilayer coating (trilayer of PDLLA, PP8020, and PP6040), where in both cases with both strains, it reached the same effect as the control of AgSD 1%, especially with 5% concentration.

### 3.7 AgSD Diffusion Study

**Figure 9:**
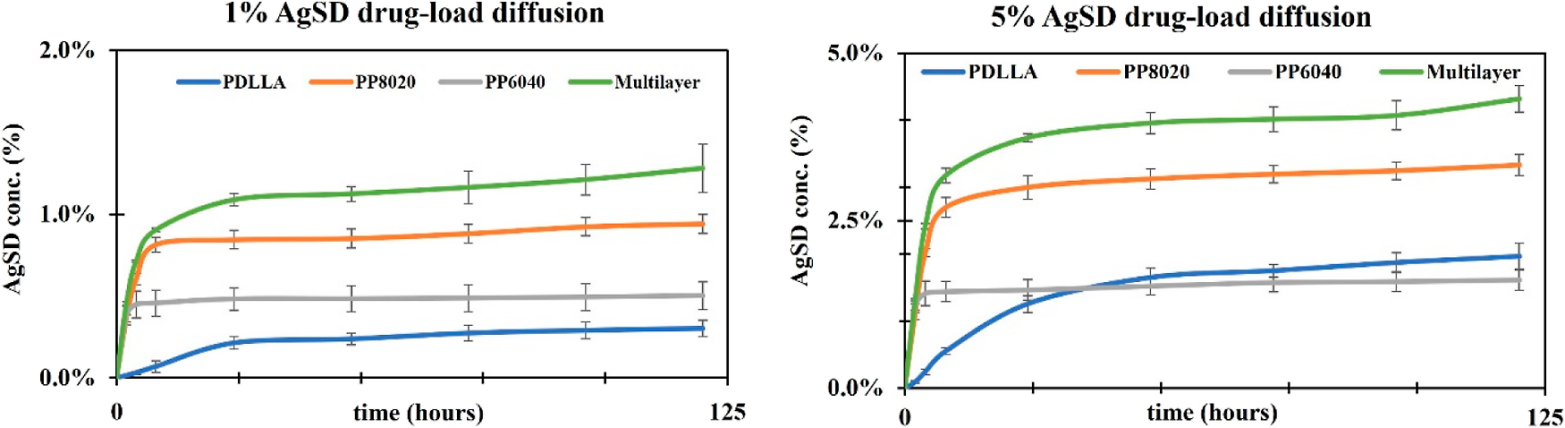
Drug diffusion study at 290 nm over 128 h in PBS at 37 °C. PDLLA (blue), PP8020 (orange), PP6040 (gray), and multilayer (green). Samples were analyzed with *n* = 3 values.

A simple drug diffusion test was performed to determine the drug release profile and approximately calculate how much AgSD was released over the period of 128 h. In Figure 9, the release profile from polymeric monolayer and multilayer coatings was assessed for AgSD (0.5–5% drug load). All the drug concentrations showed the same drug release behavior, independent of the concentration; meanwhile, the 1 and 5% diffusion profiles are shown as 1% AgSD is the standard concentration found in the commercial products and 5% is the limit that the FDA approves. Over the course of 128 h, the release of AgSD from PDLLA is not fast enough, indicating that PDLLA requires more than 120 h to fully release the drug load. Meanwhile, PEGylated versions showed better drug diffusion in the first 24 h, where PP8020 was able to achieve 0.8% drug release within 24 h when loaded with 1% AgSD and 3.0% AgSD when loaded with 5% AgSD. Coating PP6040 showed underwhelming drug loading, but the release was achieved in the first 24 h. The best results were achieved by the multilayer coating where the release is being influenced by the trilayer of PDLLA, PP8020, and PP6040, indicating that PEG addition contributes to the hydrophilicity of the material and impact increased water uptake, which increases degradation and therefore enhances the drug release over time. When multilayer approach is used, the percentage showed to increase when 1% AgSD drug load is used, this was expected as three layers are being used at 1% each; however, when drug loaded at 5%, the multilayer appeared to stay below the 5% mark at 120 h but showed signs of slightly increasing drug release after this time point. Using the AgSD calibration curve (Supporting Information: Figure S7), it was possible to estimate the drug concentration in percentage being released from the polymeric coatings.

### 3.8 Bacterial Viability

**Figure 10:**
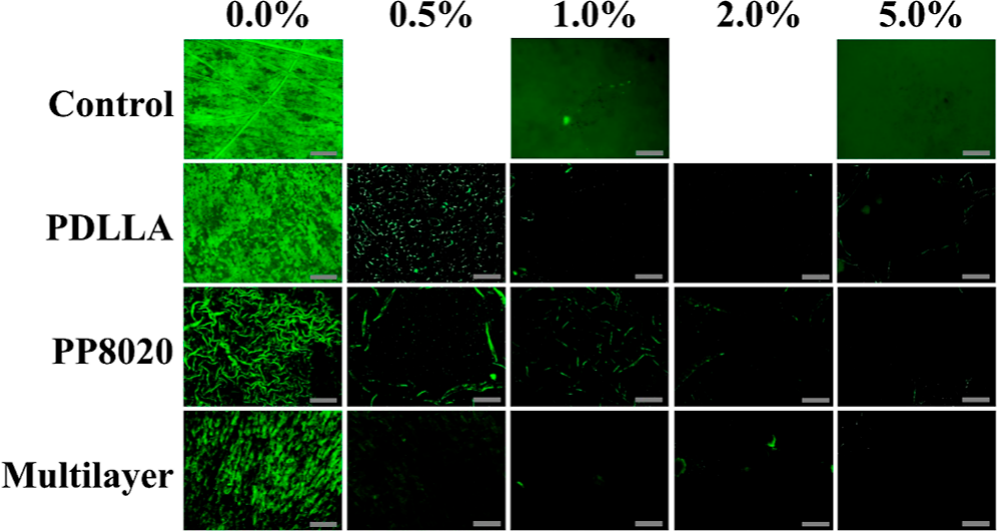
Epifluorescent microscopy of *P. aeruginosa* (PA01 + GFP) samples after the halo assay to analyze qualitatively the surface and bacterial viability, a magnification of 10× was used. Controls consist of bare titanium and filter paper with 1 and 5% AgSD. 20 μm scale bar. Samples were analyzed with *n* = 6 values.

Epifluorescent microscopy was used to further qualitatively analyze the samples from the halo assay at 10× and 60× magnification (Supporting Information: Figure S8) in order to assess the viability and confirmation of Gram-negative bacteria PA01 + GFP after being in contact with AgSD (0–5% w/v) polymeric coatings (Figure 10). Ti6Al4V was completely covered by *P. aeruginosa* after 24 h; meanwhile, for the polymeric coatings without drugs (0% AgSD), there is still bacterial adherence, but a clear decrease is seen on the biomass on the PEGylated versions, coating swelling and change of adherence to the topographical layout can also be appreciated. For AgSD, it has been reported that direct contact of AgSD >1% has shown to cause cytotoxic effects;^31^ therefore, it was expected to see that the controls of AgSD in filter paper at 1 and 5% completely eradicate the viable bacterial cells. Whereas with the polymeric coatings, we can appreciate the decrease of bacterial cells adhered to the surface due to both effects taking place in the prevention of bacterial efficacy to adhere to the surface of the coated Ti6Al4V, the antifouling effect due to the PEG addition and the antimicrobial effect due to the AgSD blend. It appears that the results from the halo assay correlate with the viability shown with the epifluorescent microscopy and fit the concentration advised for PA of >1% AgSD by the literature and the FDA.^37^

## 4 Discussion

The use of antimicrobial and antifouling coatings for medical devices, especially in the case of Ti6Al4V-based implants, shows great potential as a means of achieving the crucial goal of reducing bacterial infections by preventing biofilm formation. However, achieving an ideal coating involves a delicate balance between antimicrobial efficacy, antifouling properties, and degradation in the case of bioresorbable biomaterials. The need for proper biocompatibility and surface adhesion for successful osseointegration adds complexity to the design of these coatings. The debate between spin coating and spray coating techniques raises important considerations for the scalability and application of antimicrobial coatings. While spin coating is effective for monolayer characterization, its limitations in scale-up processes or for use on complicated 3D geometries make spray coating the preferred choice for technology transfer and industrial scale-up in real-world applications. Coating applications open different avenues as antimicrobial strategies, one of these is the difference between monolayer and multilayer coatings, which provides different combinations of polymeric layers or use of composite materials to enhance the antimicrobial effect of multilayered coatings and has been demonstrated in food and wound healing.^38−41^ While industrial applications commonly employ multilayer coatings to guarantee quality and achieve a certain finish of the coating, this study emphasizes the significance of monolayer characterization for achieving notable antifouling and antimicrobial properties against specific bacterial strains. The use of amphiphilic bioresorbable polymer coatings with varying concentrations of PEG had a clear and positive impact on the surface roughness of hydrophobic Ti6Al4V. The smooth surface achieved by certain polymers, such as PP8020 and multilayers, demonstrates their potential antifouling effect against early bacterial adhesion, which is of critical importance for biofilm prevention. The incorporation of hydrophilic PEG further enhances this effect, presenting promising results against both Gram-positive and Gram-negative bacteria that are clinically relevant. The degradation results suggested that a significant quantity of water-soluble low-molecular-weight oligomers had been generated during the first degradation stage. Mass loss started as soon as these compounds were taken out of the polymer matrix. The high-molecular-weight fraction remains insoluble; meanwhile, there is a buildup of water-soluble oligomers over time affecting the pH of the solution, which initiates an autocatalytic reaction of degradation. These findings seem to follow the model of degradation of bioresorbable polymeric discs from Farahani et al.^42^ With the contact angle results, it was possible to show the effect of PEG as a hydrophilic molecule, which is useful to modify the degree of hydrophilicity. It is still possible to control water uptake and degradability by modifying the ratios of PDLLA/PEG accordingly. The multilayer contact angle decreased slightly in comparison to the monolayer of PP6040, this was expected as PP6040 is the outermost coating in contact with the water drop, and it seems that the gradient of the different polymer coatings with different water uptake may contribute to further decrease of the contact angle.

The topography of the Ti6Al4V surface plays a major role in promoting host cell adhesion for osseointegration. However, in the presence of infections, it becomes a dual role as facilitator of bacterial adhesion contributing to the “race to the surface” between bacterial and host cells, particularly with *S. aureus* and *P. aeruginosa*.^43,44^ The vast number of grooves and wells on the surface provides ample anchoring points for biofilm formation, emphasizing the need for tailored coatings to address this complicated challenge. The relevance of MRSA and the differences in inhibition zones shed light on the complex mechanisms of antimicrobial resistance between strains involved in biofilm formation on implantable medical devices. The irregular inhibition zones of *P. aeruginosa*, coupled with increased antimicrobial effect and motility, provide insights into the variations in bacterial response to coatings.^45,46^ The effect of PEGylation enhances drug release, offering protection in the early stages of bacterial adhesion. However, optimization of the drug load is a potential avenue for further improvement. The present work acknowledges challenges with hydrophilic coatings when applied to Ti6Al4V, highlighting early detachment issues with PP6040 due to the increased water intake and uneven ratio of hydrophilicity > hydrophobicity between PP6040 and Ti6Al4V. To improve coating adhesion, the trilayer approach was taken into consideration to create a gradient of hydrophilicity between materials, diminishing the speed of detachment while preserving the gradual “peeling effect”^47^ between layers. While chemical modifications are suggested, it is emphasized that additional parameters need consideration to prevent negative effects during osseointegration, reflecting the complexity of achieving effective hydrophilic coatings on this metallic substrate.

The hydrophobic nature of AgSD poses challenges in blending with bioresorbable polymers or aqueous solutions; however, the efficacy and prevention of drug-resistance mechanisms on bacterial strains proves to be a promising antimicrobial agent. The discussion emphasizes the complexity of achieving proper drug loading with concentrations above 1% AgSD showing cytotoxic effects. This raises considerations for optimizing drug load and exploring new techniques to enhance drug addition in order to achieve a suitable prevention against bacterial adherence.

In summary, the present work provides valuable insights into the intricate balance required for developing effective antimicrobial coatings for medical devices, considering factors such as biocompatibility, coating techniques, layer strategies, topographical challenges, and the complexities associated with drug loading. Further research and optimization efforts are suggested to address the current limitations and enhance the efficacy of these coatings.

## 5 Conclusions

As previously discussed, this development marks a major leap in the effectiveness and versatility of antibacterial coatings for hard-tissue implants. Therefore, the bioresorbable amphiphilic coatings produced have smoother implant surfaces with less surface roughness, which is important for the prevention of bacterial adherence, especially against *S. aureus* and *P. aeruginosa*. Moreover, PEG hydrophilicity combined with bioresorbability and biocompatibility of aliphatic polyesters offers a potentially effective way to address issues with permanent antimicrobial coatings on Ti6Al4V implants where surface modifications are often required and can have detrimental effects. Bioresorbable materials can alleviate challenges regarding further surface modifications and possible osseointegration disruptions, as these materials can shine especially during the wound healing process, where bacterial infections are more prone to happen.

The effects of PEG when combined with other elements, such as AgSD and amphiphilic polymers, result in enhanced and controlled drug release, protecting medical devices against the early attachment of pathogens. The suggestion for optimizing the drug load further emphasizes the potential for continuous future improvement and fine-tuning of these hydrophilic coatings for maximum coverage and protection. Bioresorbable antimicrobial coatings have a positive effect that is evident in their increased biocompatibility, decreased bacterial adhesion, and enhanced drug release capabilities. These coatings not only solve issues related to coating detachment and bacterial proliferation but also open new possibilities for the development of sophisticated antimicrobial coatings. The promising results presented in this work highlight the potential of amphiphilic bioresorbable polymers as antimicrobial and antifouling coatings, paving the way for the next generation of hydrophilic coatings for Ti-based hard-tissue implants.

## Supporting Information Available

^1^H NMR spectra in DMSO-*d*_6_ (2.50) and comparison of d,l-lactide, PDLLA, PDLLA–PEG (80:20 and 60:40), and mPEG_5000_, with ‘e’ peak (4.6–4.4) showing the hydroxyl group from mPEG, suggesting successful copolymerization; GPC-THF curves of PDLLA, PP8020, PP6040, and mPEG_5000_; pH monitoring overtime (49 days) of PDLLA, PDLLA–PEG 80:20, and PDLLA–PEG 60:40; structural integrity of PDLLA, PP8020, and PP6040 over the degradation time; SEM surface analysis for half-coated disc to identify through SEM titanium and how homogeneous was the polymeric coating; polynomial second-order images used to remove background for easier bacterial identification; calibration curve of AgSD in PBS for 0.01–5.00% w/v of antimicrobial; and epifluorescent microscopy of *P. aeruginosa* (PA01 + GFP) samples after the halo assay at 60× magnitude (PDF)

## Abbreviations

^1^H NMR: proton nuclear magnetic resonance
AFM: atomic force microscopy
AgSD: silver sulfadiazine
BRP: bioresorbable polymer
CDCl_3_: deuterated chloroform
CHCl_3_: chloroform
DMSO-*d*_6_: deuterated dimethyl sulfoxide
DLA: d,l-lactide
DMSO: dimethyl sulfoxide
EDS: energy-dispersive spectroscopy
kDa: kilodaltons
LA: lactide
*M*_n_: number-average molecular weight
mPEG: methyl poly(ethylene glycol)
*M*_w_: weight-average molecular weight
PBS: phosphate buffered saline
PDI: polydispersity index
PDLLA: poly(d,l-lactide)
PDLLA-mPEG: poly(d,l-lactide)—b—methyl ether poly(ethylene glycol)
PEG: poly(ethylene glycol)
ROP: ring-opening polymerization
RT: room temperature
SEM: scanning electron microscopy
Sn (Oct)_2_: stannous octoate
THF: tetrahydrofuran
*W*_d_: dry mass
*W*o: initial mass
*W*_t_: wet mass
