# Supplementary material for: Bioresorbable Polyester Coatings with Antifouling and Antimicrobial Properties for Prevention of Biofilm Formation in Early Stage Infections on Ti6Al4V Hard-Tissue Implants
Source: ACS Appl Bio Mater. 2024 Jul 22;7(8):5728–39. doi: 10.1021/acsabm.4c00832 (PMC11337155; doi:10.1021/acsabm.4c00832)
Supplement: Supplementary file 1 — mt4c00832_si_001.pdf [file mt4c00832_si_001.pdf]

SUPPORTING INFORMATION

Bioresorbable polyester coatings with antifouling and antimicrobial properties for prevention of biofilm formation in early-stage infections on Ti6Al4V hard-tissue implants.

David Zermeño Pérez[1,4, *], Hamza Chouirfa[1], Brian J. Rodriguez[2], Thomas Dürig[3], Patrick Duffy[1], Tadhg Ó Cróinín[4, *].

[1]Ashland Specialties Ireland Ltd., Mullingar, Ireland

[2]School of Physics, University College Dublin, Dublin, Ireland

[3]Ashland Wilmington Centre, Delaware, U.S.A.

[4]School of Biomolecular and Biomedical Science, University College Dublin, Dublin, Ireland

*Correspondence: david.zermenoperez@ashland.com / tadhg.ocroinin@ucd.ie

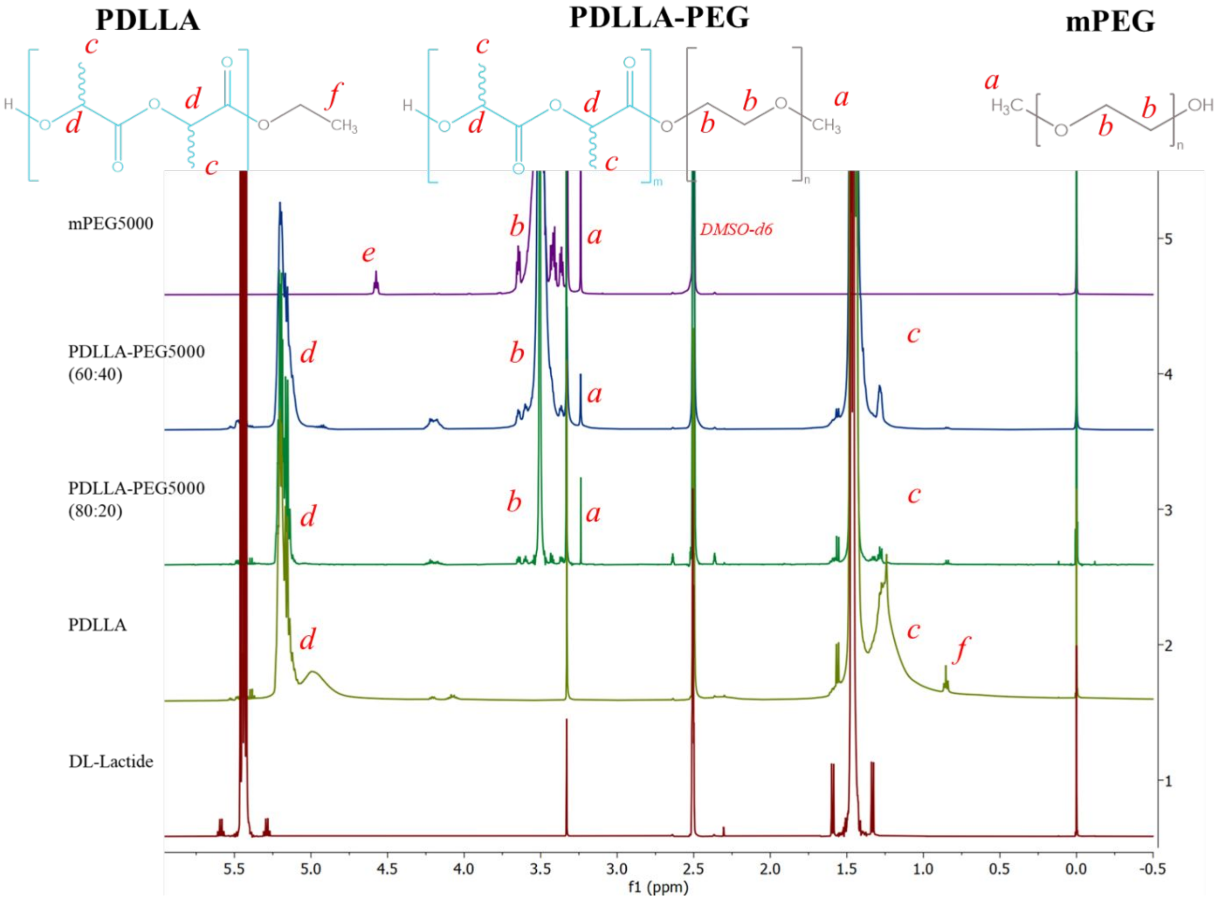

Figure S1. ^{1}H-NMR spectra in DMSO-d6 (2.50); comparison of *D,L*-Lactide, PDLLA, PDLLA-PEG (80:20 and 60:40) and $mPEG_{5000}$. '*e*' peak (4.6-4.4) showing the hydroxyl group from mPEG, suggesting successful co-polymerization.

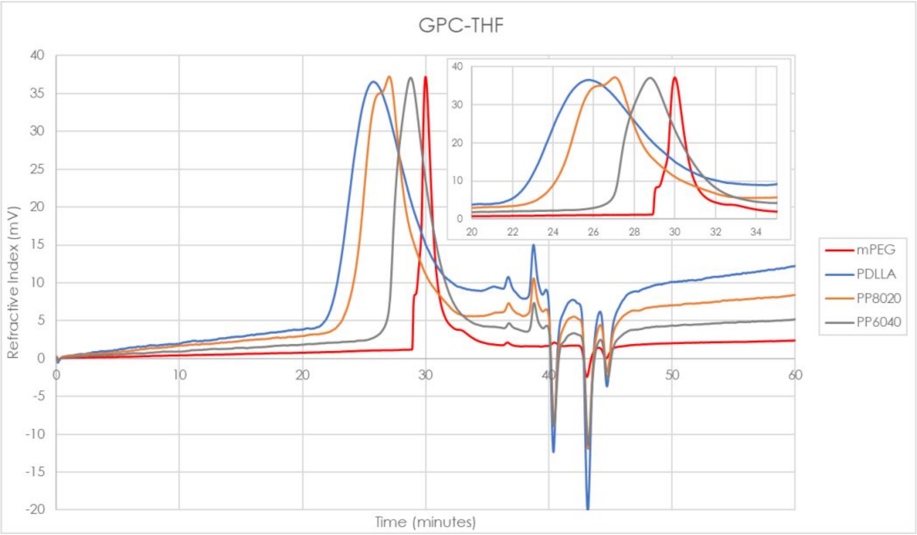

Figure S2. GPC-THF curves of PDLLA (blue), PP8020 (orange), PP6040 (grey) and $mPEG_{5000}$ (red). All peaks were adjusted to the same height for comparative purposes. One population is seen in all samples, except in PP20 where a slight shoulder can be appreciated. Blue (PDLLA), orange (PDLLA-PEG 80:20) and grey (PDLLA-PEG 60:40).

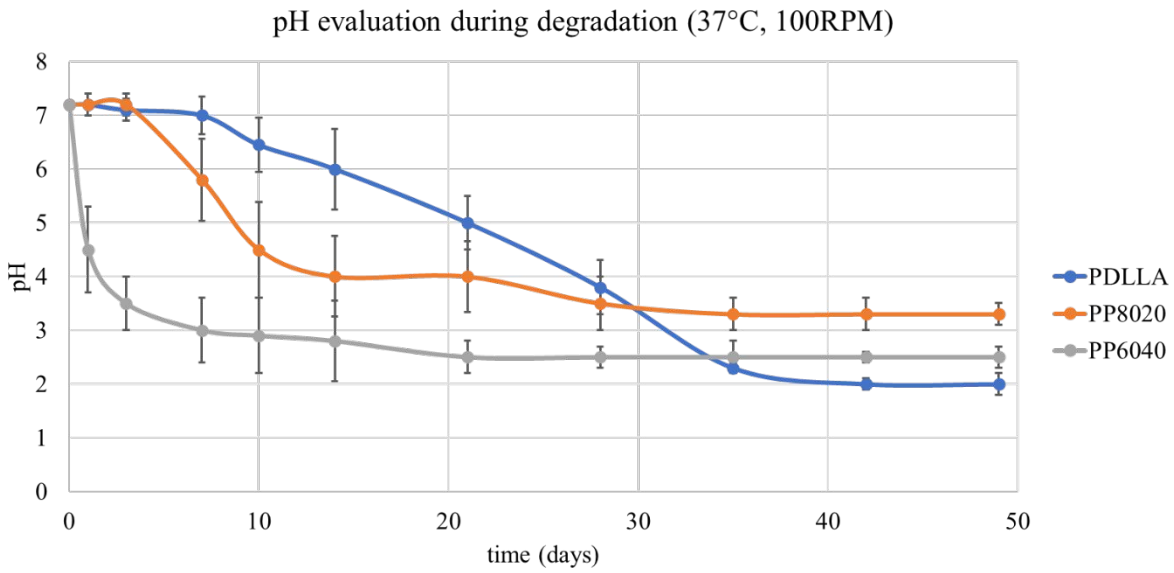

Figure S3. pH monitoring overtime (49 days). Blue (PDLLA), orange (PDLLA-PEG 80:20) and grey (PDLLA-PEG 60:40).

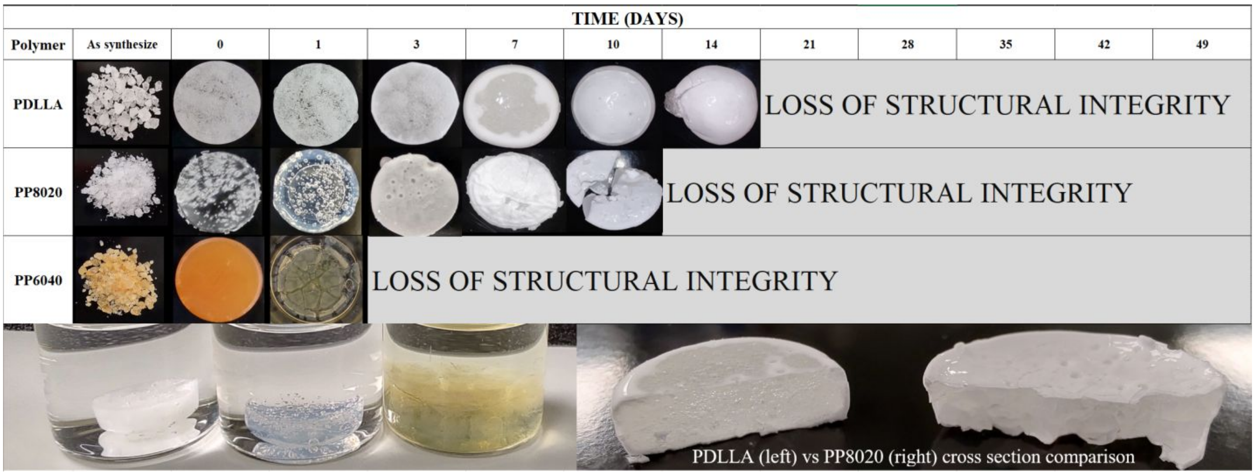

Figure S4. Structural integrity of PDLLA, PP8020 and PP6040, over the degradation time. Swelling behavior and porosity increase after 3 days after drying.

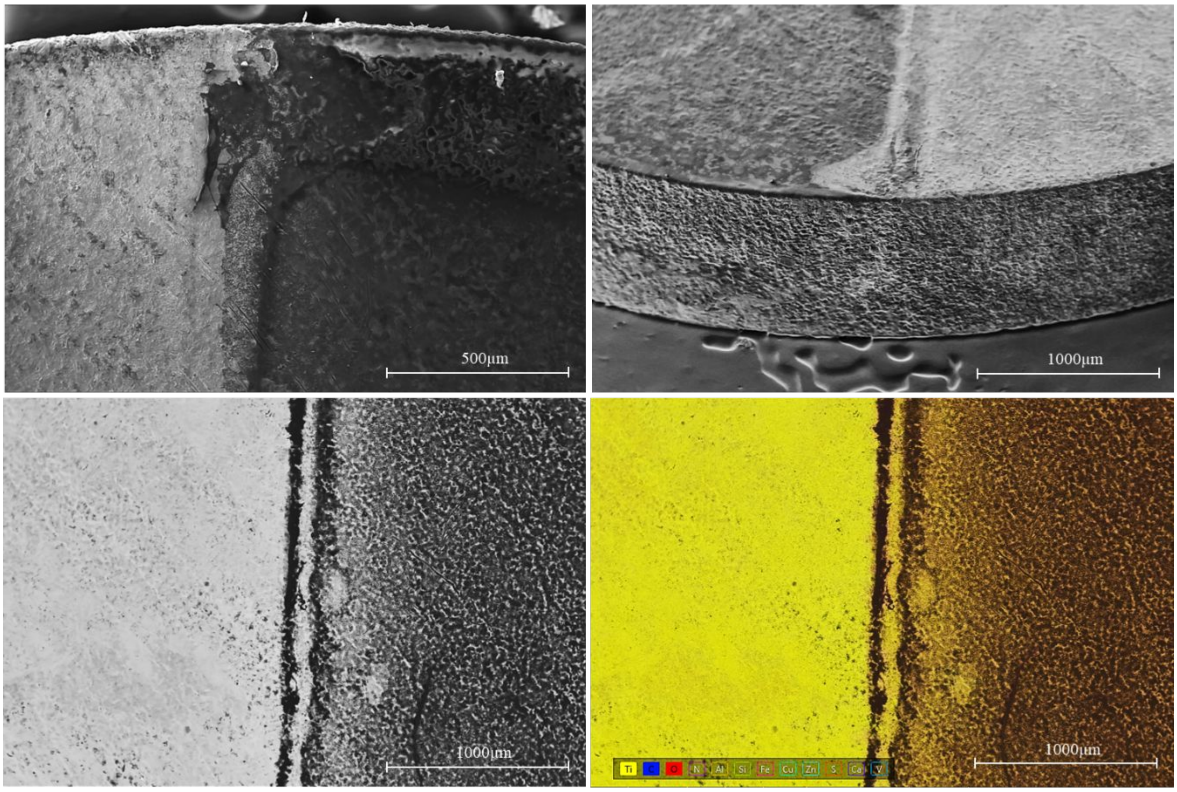

Figure S5. SEM surface analysis for half coated disc to identify through SEM titanium (yellow) and how homogeneous was the polymeric coating (C in blue and O in red)

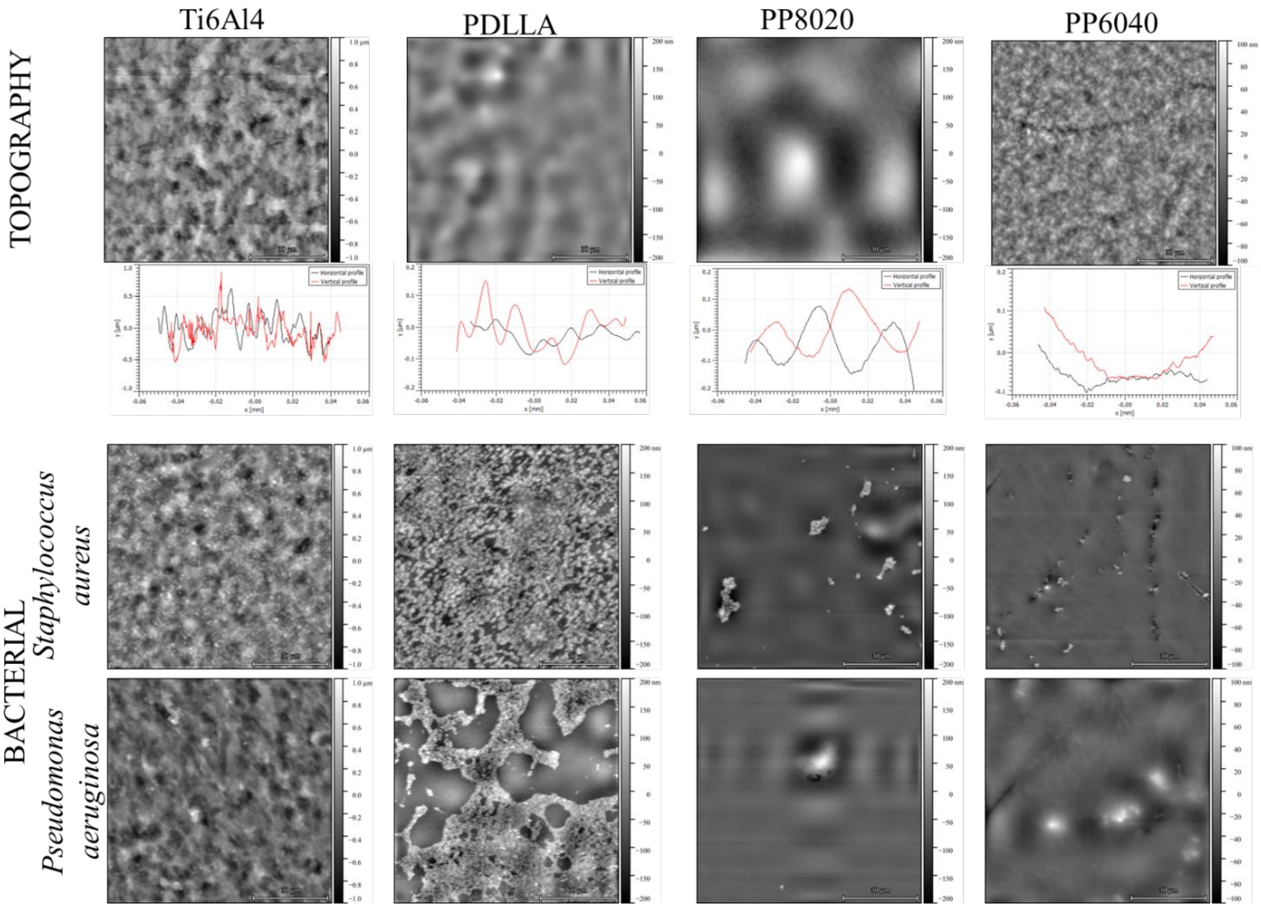

Figure S6. polynomial 2nd order images used to remove background for easier bacterial identification.

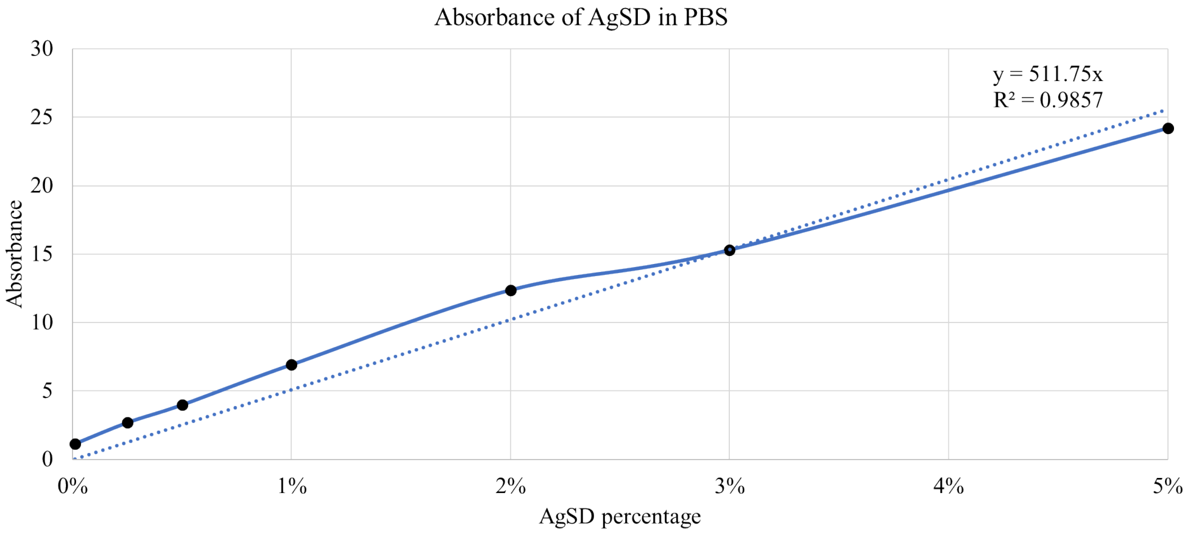

Figure S7. Calibration curve of AgSD in PBS for 0.01 to 5.00% w/v of antimicrobial.

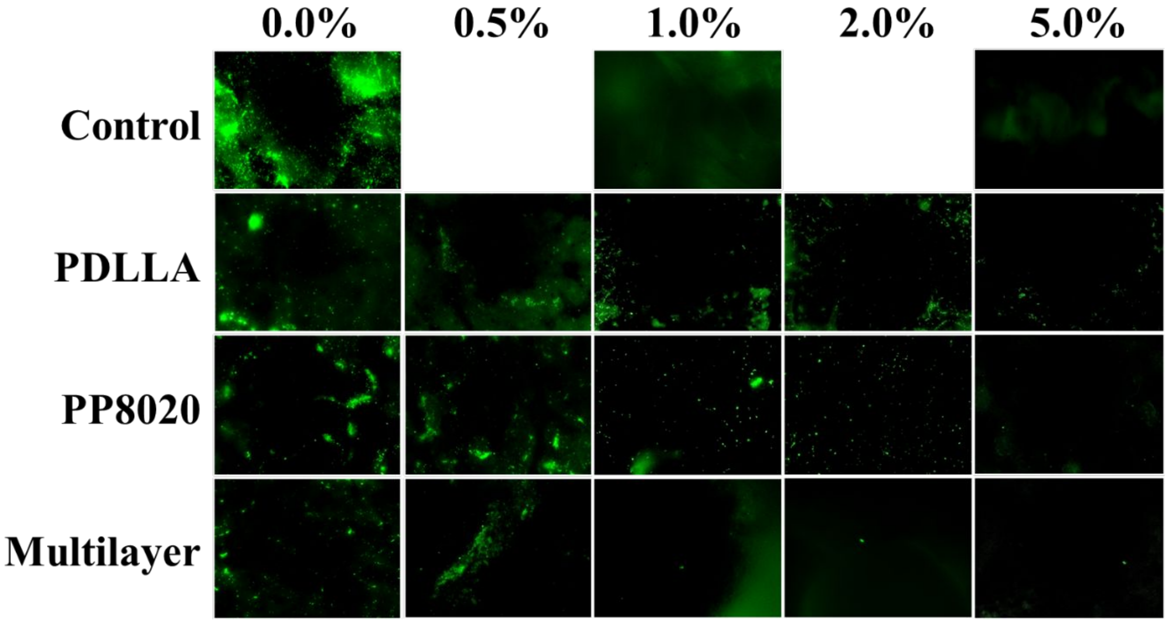

Figure S8. Epifluorescent microscopy of *Pseudomonas aeruginosa* (PA01+GFP) samples after halo assay, to analyze presence of bacterial cells on the surface, magnification of 60x was used. Controls consist of bare titanium and filter paper with 1% and 5% AgSD.
